# Age-associated changes in the immune system may influence the response to anti-PD1 therapy in metastatic melanoma patients

**DOI:** 10.1007/s00262-020-02497-9

**Published:** 2020-02-08

**Authors:** Henna Kasanen, Micaela Hernberg, Siru Mäkelä, Oscar Brück, Susanna Juteau, Laura Kohtamäki, Mette Ilander, Satu Mustjoki, Anna Kreutzman

**Affiliations:** 1 Hematology Research Unit Helsinki, Department of Clinical Chemistry and Hematology, University of Helsinki and Helsinki University Hospital Comprehensive Cancer Center, Haartmaninkatu 8, 00290 Helsinki, Finland; 2 Translational Immunology Research Program, University of Helsinki, Helsinki, Finland; 3 Department of Oncology, Helsinki University Hospital Comprehensive Cancer Center, Helsinki, Finland; 4 iCAN Digital Precision Cancer Medicine Flagship, University of Helsinki, Helsinki, Finland; 5 Department of Pathology, Haartman Institute, University of Helsinki, Helsinki, Finland

**Keywords:** Immunotherapy, Anti-PD1, Chemokines, NKT cells, Biomarkers, Age

## Abstract

Anti-PD1 treatment has improved the survival of metastatic melanoma patients, yet it is unknown which patients benefit from the treatment. In this exploratory study, we aimed to understand the effects of anti-PD1 therapy on the patients’ immune system and discover the characteristics that would result in successful treatment. We collected peripheral blood (PB) samples from 17 immuno-oncology-naïve metastatic melanoma patients before and after 1 and 3 months of anti-PD1 therapy. In addition, matching tumor biopsies at the time of diagnosis were collected for tissue microarray. The complete blood counts, PB immunophenotype, serum cytokine profiles, and tumor-infiltrating lymphocytes were analyzed and correlated with the clinical data. Patients were categorized based on their disease control into responders (complete response, partial response, stable disease > 6 months, *N* = 11) and non-responders (progressive disease, stable disease ≤ 6 months, *N* = 6). During therapy, the PB natural killer T (NKT) cell frequency, expression of CD25 and CD45RO on cytotoxic natural killer (NK) cells, and serum CXC chemokine levels were significantly increased in responders. Furthermore, higher age together with age-associated characteristics from PB, lower frequency of PB-naïve CD8^+^ T cells, and elevated levels of serum MCP-4 and OPG were discovered as baseline predictors of treatment response. We therefore propose that in addition to T cells, anti-PD1 treatment is associated with NK- and NKT-cell population dynamics, and that the age-associated characteristics from PB together with older age may contribute to prolonged PFS in anti-PD1-treated melanoma patients.

**Electronic supplementary material:**

The online version of this article (10.1007/s00262-020-02497-9) contains supplementary material, which is available to authorized users.

## Introduction

Immunotherapies have come to stay in the treatment of advanced melanoma due to their improved efficacy that has translated into superior overall survival compared to conventional therapies such as chemotherapy [1]. From the recent plethora of novel immune therapies, the checkpoints inhibiting monoclonal antibodies, anti-PD1 and anti-CTLA4 have been promising in the treatment of metastatic melanoma [2, 3], whereas many other are currently undergoing early clinical trials [4]. The inhibition of the PD1 pathway reactivates the anti-tumor T-cell response by blocking the inhibitory signal induced by tumor cells [5]. However, no comprehensive immunomonitoring of the patients during anti-PD1 has been performed. Especially, the in vivo effects of anti-PD1 on other lymphocytes than T cells, such as natural killer (NK) and natural killer T (NKT) cells in cancer patients remain unexplored.

NK cells are key effectors in the first line immune defense due to their rapid response to virally infected and tumor cells. Ligands that bind to NK-cell-activating receptors are typically overexpressed in tumors, whereas in healthy cells, these activating ligands are not as abundant [6]. Further, the expression of major histocompatibility complex (MHC) class I may be downregulated in tumor cells, enabling them to evade CD8^+^ T cells and become more sensitive to NK cells [7]. NKT cells are a group of T cells that have shared properties of both NK and T cells. When activated, NKT cells produce large amounts of cytokines, such as interferon-γ (IFN-γ), which adjust the activation of NK and T cells [8, 9].

To maintain balance in the immune system, the functions of T, NK and NKT cells are regulated via several mechanisms, such as with the complex cytokine network, based on receptor–ligand interactions. In cancer, cytokines have both pro- and anti-tumor properties [10]. For example, chemotactic cytokines, CXCL9 and CXCL10 may promote anti-tumor immune responses by recruiting NK and T cells into the tumor tissue thereby inhibiting angiogenesis. Conversely, CXCL16 enhances the tumor-promoting immune responses in macrophages [11]. In addition to soluble cytokines, the regulation of their corresponding cell surface receptors is crucial for controlling immune system activation. For example, interleukins (IL) 12, 18 and 21 are known to induce NK-cell cytotoxicity once bound to receptors on the NK-cell surface [12].

During recent years, anti-PD1 has remarkably improved the survival of melanoma patients. In a phase 3 controlled study, anti-PD1-treated metastatic melanoma patients without prior treatment had significantly improved overall (OS) and progression-free survival (PFS) compared to chemotherapy-treated patients [13]. Anti-PD1 treatment was also proven to be more effective and less likely to cause severe adverse events (AEs) compared to chemotherapy in the treatment of advanced melanoma [14, 15]. However, it is yet unknown why only some patients benefit from anti-PD1 therapy.

In this exploratory study, we investigated the effects of anti-PD1 therapy on the immune system of metastatic melanoma patients before and after 1 and 3 months of therapy. Our primary objective was to determine temporal changes occurring during PD1 inhibition in T-, NK and NKT cells, which all play a key role in tumor suppression and elimination [16, 17]. Next, we sought to delineate the immune and clinical characteristics between the patients who were responsive to the treatment compared to those that did not, to find a potential biomarker of response.

## Materials and methods

### Patients and samples

The study included 17 metastatic melanoma patients treated with anti-PD1 therapy; nivolumab (Bristol-Myers Squibb) or pembrolizumab (Merck) (patient characteristics in Table 1) at the Helsinki University Hospital Comprehensive Cancer Center. Samples were collected within a 2-year time frame from all volunteering patients who had not received any prior immuno-oncological (IO) therapy. None of the patients had prior autoimmune diseases and The Eastern Cooperative Oncology Group performance status was scored either 0 or 1. The metastasis stage was evaluated using the AJCC7 staging manual [18], and responses were evaluated using the RECIST criteria [19].

**Table 1:** Patient characteristics

	Responders	Non-responders
Patients		
* N*	11	6
Age		
Median	71.0 (66–84)	62.5 (53–78)
Sex		
Female	5 (45%)	2 (33%)
Male	6 (55%)	4 (67%)
Metastasis		
M1a	4 (36%)	–
M1b	3 (27%)	1 (17%)
M1c	4 (36%)	5 (83%)
BRAFV600		
Mutated	1 (17%)	3 (50%)
No mutation	10 (83%)	3 (50%)
PreLDH		
Median	240.0 Ul (159–357)	226.5 Ul (180–411)
Drug		
Nivolumab	4 (36%)	3 (50%)
Pembrolizumab	7 (64%)	3 (50%)
Follow-up time		
Median	21.0 months (12–27)	21.0 months (12–27)
PFS		
Median	12.0 months (3–20)	3.0 months (1–6)
Best response		
CR	2 (18%)	–
PR	5 (45%)	–
SD	4 (36%)	1 (17%)
PD	–	5 (83%)

*M1a* metastasis in distant skin sites or areas under the skin or in distant lymph nodes with normal LDH, *M1b* metastasis in lung with normal LDH levels, *M1c* metastasis in internal organ or any other metastasis with elevated LDH. Best achieved response during anti-PD1 therapy: *CR* complete response, *PR* partial response, *SD* stable disease, *PD* progressive disease

Peripheral blood (PB) samples were collected at three time points: before initiation (pre), after 1 (1mo) and 3 months (3mo) of anti-PD1 treatment (sampling and drug infusion schedule is illustrated in supplemental Fig. 1). Complete blood counts (CBC) were performed concurrently with the study samples during routine clinical tests. Nationally evaluated values obtained from the HUSLAB laboratories were used as a reference. In addition, formalin-fixed paraffin-embedded (FFPE) tumor samples from primary and metastatic melanoma biopsies that were taken during the time of diagnosis before IO therapy were collected. Furthermore, PB samples from ten healthy volunteers were collected as controls.

### Immunophenotyping of peripheral blood

The lymphocyte subpopulations were immunophenotyped from fresh PB samples for various cell surface markers, including immune checkpoint receptors, markers for chemotaxis, cytotoxicity, and migration. The panel is presented in supplemental Table 1. 50,000 CD45^+^ lymphocytes were acquired with FACS Verse (BD) and the data were analyzed with FlowJo (FlowJo 10.4, FlowJo, LLC 2006–2017).

### Serum protein analysis

Serum samples separated from fresh PB using centrifugation were stored in − 70 °C. The samples were analyzed with a proximity extension assay (Proseek Multiplex Inflammation panel, Olink Bioscience). The samples were run on two separate plates and duplicate samples were used to normalize the differences between the two runs. Protein levels were expressed as Normalized Protein eXpression (NPX) values, an arbitrary log_2_-scale unit.

### Tissue microarray (TMA) and multiplexed immunohistochemistry (mIHC)

FFPE metastatic melanoma tumor biopsies at the time of diagnosis, within 3 months to 1 year before initiation of anti-PD1 therapy (*n* = 9, six from responders, three from non-responders), were used. Tissue core punches 2 mm in diameter were collected from the area containing cancerous cells to construct the TMA block. Sections from the TMA block were used for mIHC to stain the tumor-infiltrating lymphocytes (TILs) as used previously [20]. The panel included markers for CD3, CD4, CD8, CD25, CD56, GranzymeB, LAG3 and PD1.

### Statistical analysis and data visualization

Statistical analysis and visualization were done with RStudio (Version 1.1.383) and GraphPad Prism (Version 8.0.1 (145)). Mann–Whitney *U* test was used to compare the ranks between the responders (R) and non-responders (NR) (two-tailed, unpaired) and Student’s *t* test to examine the significance between paired observations at different time points (before initiation of anti-PD1 vs. after 1 or 3 months of therapy). When comparing more than two groups, Kruskal–Wallis and Dunn’s multiple comparison tests were used. Due to the limited number of patients, no correction for multiple testing was performed. The range of *P* values are labeled with asterisks (*P < 0.05, **P < 0.01, ***P < 0.001).

## Results

### Patients’ characteristics

Our cohort consisted of 17 IO-naïve metastatic melanoma patients treated with anti-PD1 monotherapy. The patients were divided based on their treatment response as responders [R, complete response (CR, *n* = 2), partial response (PR, *n* = 5) or stable disease (SD, *n* = 4) > 6 months] and non-responders [NR, SD ≤ 6 months, *n* = 1, or progressive disease (PD, *n* = 5)]. The median age of the responders was 71 and non-responders 62.5 years (*p* = 0.04). All characteristics of the responder and non-responder cohorts are summarized in Table 1.

Patients were randomly selected for treatment with nivolumab (*n* = 7) or pembrolizumab (*n* = 10). Overall, 64% of the responders and 50% of the non-responders received pembrolizumab. All patients were monitored for follow-up at least 12 months from the initiation of therapy with a median of 21 months. The median PFS of the responders and non-responders was 12.0 and 3.0 months, respectively.

### Patients responding to PD1 inhibition have higher quantities of circulating lymphocytes during treatment

To first assess the treatment effects on the leukocyte subpopulations, the frequencies and absolute values of the CBCs were analyzed before and during therapy.

**Fig. 1:**
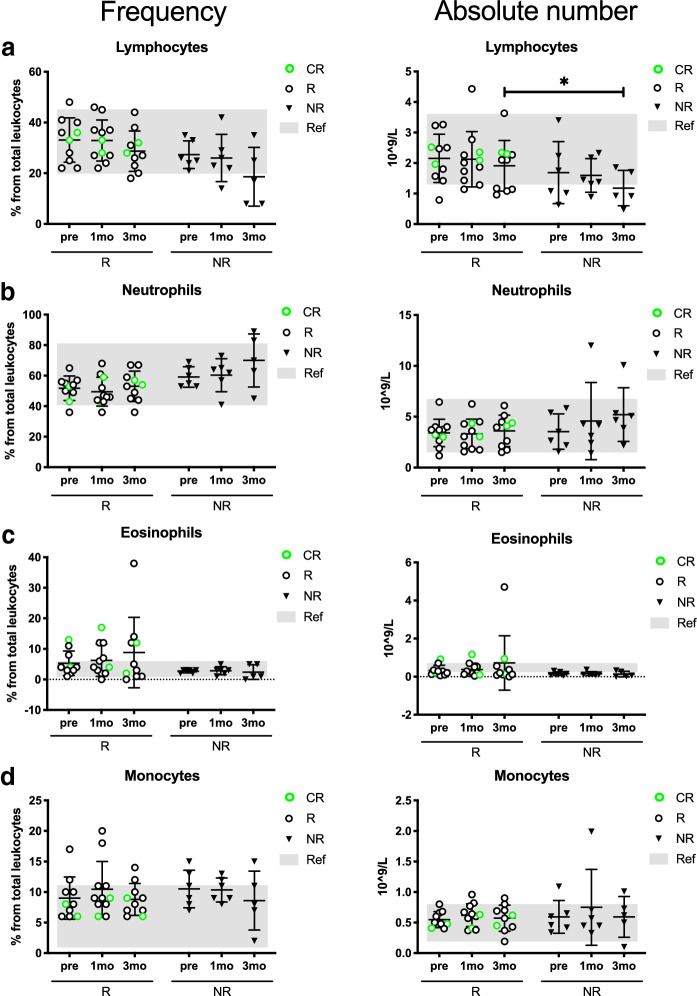
Patients responding to PD1 inhibition have higher peripheral blood lymphocytes during treatment. Frequency and absolute count of **a** lymphocytes, **b** neutrophils, **c** eosinophils and **d** monocytes from peripheral blood samples of anti-PD1-treated metastatic melanoma patients before and during treatment. Gray areas indicate the normal reference range (Helsinki University Hospital HUSLAB laboratories), green circles represent complete responders (CR), black circles represent responders (R), and black triangles represent non-responders (NR). The statistical difference between R and NR cohorts are calculated with the Mann–Whitney *U* test

The absolute lymphocyte count was significantly higher in the responders (*R*_mean_: 1.9 10^9^/L vs. NR_mean_: 1.2 10^9^/L, *p* = 0.04) after 3 months of anti-PD1 treatment (Fig. 1a). The number and proportion of the lymphocytes seemed to decrease in all patients, but particularly in the NR group during therapy. No statistically significant differences were observed in other leukocyte populations, except for a trend in lower neutrophil frequencies in the responders after 3 months of anti-PD1 therapy compared to non-responders (*R*_mean_ 53.5% vs. NR_mean_ 70.0%, *p* = 0.06) (Fig. 1b). In addition, the eosinophil frequencies in a proportion of the responders was higher than the normal reference range (*R*_mean_ 8.8%, normal reference range 1.0–6.0%) after 3 months of anti-PD1 therapy (Fig. 1c). No significant differences were observed in the monocyte populations (Fig. 1d). The neutrophil (NLR), eosinophil (ELR) and monocyte (MLR) to lymphocyte ratios did not indicate statistically significant differences between responders and non-responders.

### The responders have high frequency of PB NKT cells before initiation and during anti-PD1 treatment

**Fig. 2:**
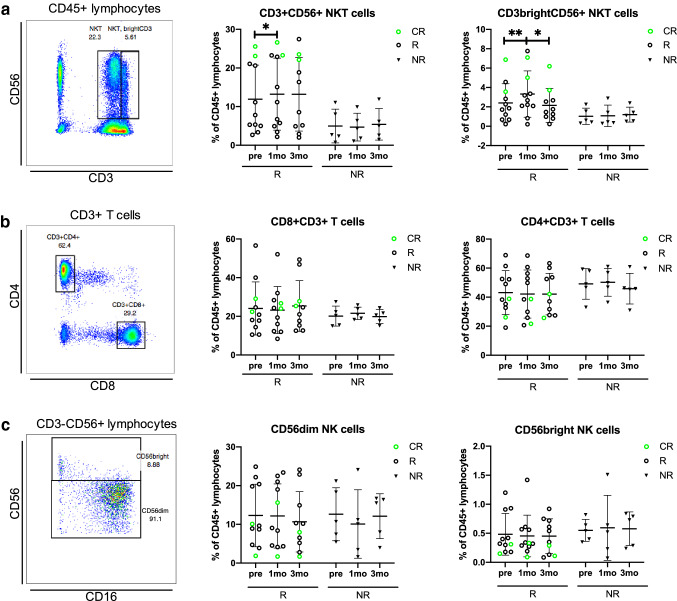
The proportion of NKT cells increases in responders after 1 month of anti-PD1 treatment. Gating strategies of **a** CD3^+^CD56^+^ and CD3brightCD56^+^ NKT cells from lymphocytes, **b** CD3^+^CD4^+^ and CD3^+^CD8^+^ T cells from total T cells, **c** CD56dim and CD56bright NK cells from the total lymphocyte population before treatment (pre), at 1 month (1mo) and at 3 months (3mo) of anti-PD1 therapy. Green circles represent the complete responders (CR), black circles represent responders (R) and black triangles represent non-responders (NR). The statistical difference between time points within same cohort was calculated with Students *t* test

To study the effects of anti-PD1 treatment on the lymphocytes, fresh PB samples were immunophenotyped before and during therapy. A representative example including the gating strategy is presented in Fig. 2.

After 1 month of anti-PD1 therapy, the mean proportion of the total NKT cells (*R*: pre 11.9% vs. 1mo 13.2%, *p* = 0.01) and CD3^bright^ NKT cells (*R*: pre 2.4% vs 1mo 3.3%, *p* = 0.007) increased in the responders, but not in the non-responders. The mean proportion of the responders’ NKT CD3^bright^ T cells later decreased back to the pretreatment levels after 3 months of anti-PD1 therapy (3.3% vs. 2.2%, *p* = 0.02). Two patients who achieved complete response had the highest total NKT-cell frequencies before and during therapy (CR_mean_ vs. other *R*_mean_; Pre: 24.5% vs. 9.1%, 1mo: 25.0% vs. 10.6%, 3mo: 23.0% vs. 10.8%, Fig. 2a). Though not significant, the responders displayed a trend in higher mean proportions of PB NKT cells (*R*_mean_ 11.2% vs. NR_mean_ 4.3%, *p* = 0.07) after 1 month of anti-PD1 treatment, compared to non-responders. No significant changes in T- or NK-cell subsets in the responders and non-responders were observed during anti-PD1 therapy (Fig. 2b, c).

### Responders’ T- and NK-cell immunophenotype differs from non-responders and healthy already before anti-PD1 therapy

**Fig. 3:**
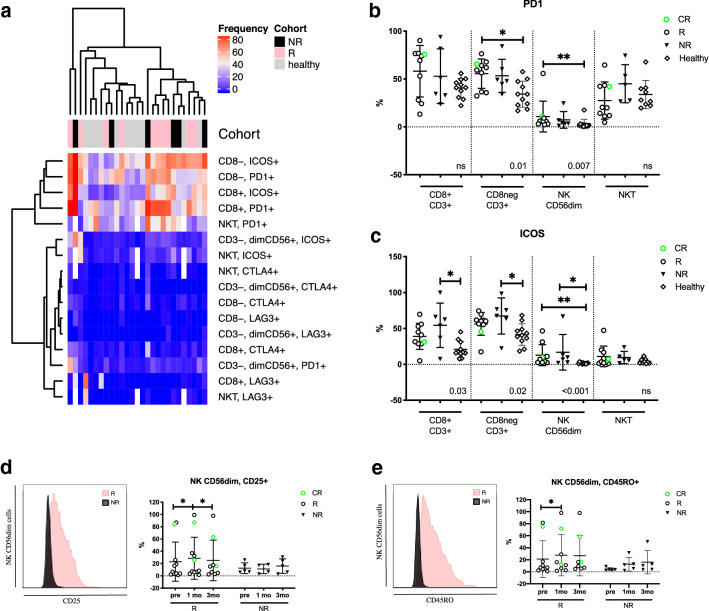
Pretreatment expression of immune checkpoint markers on T, NKT and NK cells. **a** Heatmap of checkpoint marker expression on healthy and patient T, NKT and NK cells before initiation of anti-PD1 therapy, clustering done using Euclidean distance. Annotation bar indicating cohorts; black for non-responders (NR), pink for responders (R) and gray for healthy. **b** PD1 and **c** ICOS expression on CD3^+^CD8^+^ and CD3^+^CD8^−^ T cells, NK cells and NKT cells at pretreatment stratified by response to anti-PD1 treatment and healthy controls. **d** CD25 and **e** CD45RO expression visualized with a density histogram and with dot plot grouped by treatment response and time point. Green circles represent the complete responders (CR), black circles represent responders (R), black triangles represent non-responders (NR), diamonds represent healthy controls (healthy). *p* values in **b** and **c** were calculated with Kruskal–Wallis ANOVA; the range of *p* values from Dunn’s multiple comparisons test are labeled with asterisks. The statistical difference between time points within the same cohorts (**d**, **e**) was calculated with the Students *t* test

To investigate the immune checkpoint profiles on lymphocytes, we compared the proportion of PD1+, ICOS+, LAG3+ and CTLA4+ expressions on the immune cells (Euclidean distance). The expression of immune checkpoint markers on the healthy controls’ and patients’ immune cells before initiation of anti-PD1 therapy is illustrated in the heatmap (Fig. 3a). The expression of PD1 and ICOS on T and NKT cells was more abundant compared to other immune checkpoint receptors, CTLA4 and LAG3, which were scarcely or not at all expressed in both the patient and healthy immune cells. No distinct clustering was observed between the cohorts, but PD1 and ICOS appeared to be more frequently expressed on the patients’ lymphocytes than in the healthy (Fig. 3a). Further, the expression of PD1 was significantly higher on the responders’ CD8^−^CD3^+^ T and CD56^dim^ NK cells than in the healthy controls (mean: CD8^−^CD3^+^ 55.6% vs. 34.2%, *p* = 0.01, CD56^dim^ NK: 10.7% vs. 3.1%, *p* = 0.007, respectively) (Fig. 3b). In contrast, the pretreatment ICOS expression was significantly higher in the non-responders’ CD8^+^CD3^+^ T, CD8^−^CD3^+^ T, and CD56^dim^ NK cells, as well as the responders’ CD56^dim^ NK cells when compared to the healthy controls (mean: CD8^+^CD3^+^ 54.4% vs. 20.9%, *p* = 0.03, CD8^−^CD3^+^ 67.4% vs. 41.5%, *p* = 0.02, NR CD56^dim^ NK 16.9% vs. 1.7%, *p* = 0.01, R CD56^dim^ NK 12.8% vs. 1.7%, *p* = 0.002, respectively) (Fig. 3c).

The NK-cell phenotyping indicated that the receptors CD45RO and CD25 were more frequently expressed in a proportion of the responders’ CD56^dim^ NK cells, but no significant differences between responders and non-responders were observed. However, the expression of both markers on the responders’ CD56^dim^ NK cells was significantly increased after 1 month of therapy (CD25: pre 23.0% vs. 1mo 28.6%, *p* = 0.03, CD45RO: pre 21.1% vs. 1mo 27.6%, *p* = 0.04), and after 3 months, the CD25 expression moderately decreased toward pretreatment levels (1mo 28.6% vs. 3mo 25.1%, *p* = 0.04) (Fig. 3d, e).

### Anti-PD1 therapy increases plasma CXCR3 ligands and lymphocyte-activating chemokines in the blood

To further study the effects of anti-PD1 therapy on the immune system, we performed a proximity extension assay to detect 92 different serum proteins before and during therapy. In addition, sera from ten healthy donors were analyzed as reference controls.

**Fig. 4:**
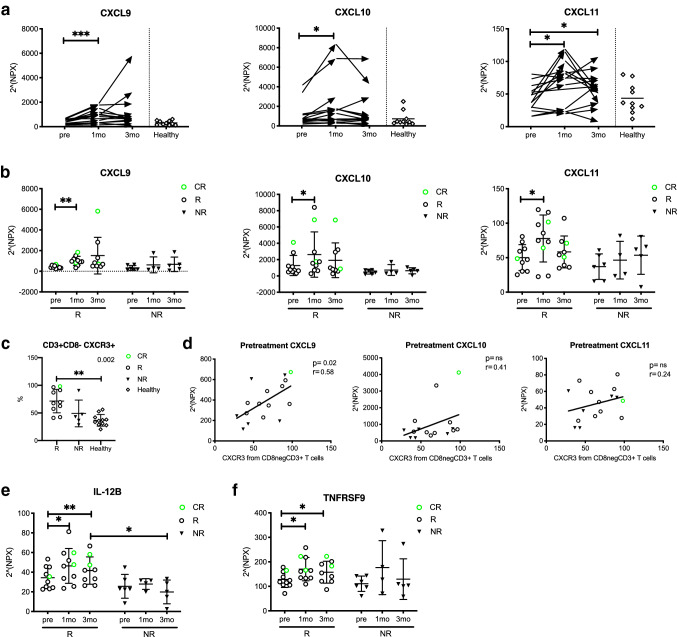
Cytokines related to antitumor immune response increase in responders’ peripheral blood during anti-PD1 treatment. Cytokine levels of CXCL9, CXCL10 and CXCL11 in **a** all patients at different time points and **b** in responders (R) vs. non-responders (NR) measured with the Olink immunoassay. **c** Proportion of CXCR3^+^ CD3^+^CD8^−^ T cells at pretreatment defined by flow cytometry in R, NR and healthy subjects. **d** Correlation of CXCR3^+^ CD3^+^CD8^−^ T cells and serum CXCL9, CXCL10 and CXCL11. **e** Cytokine levels of IL-12B and **f** TNFRSF9 in R and NR. Protein levels are presented as Normalized Protein eXpression (NPX) values, an arbitrary unit on the log_2_-scale. Green circles represent the complete responders (CR), black circles represent responders (R) and black triangles represent non-responders (NR), diamonds represent healthy controls (healthy). Statistical differences between time points within the same cohort were calculated with the Student’s *t* test (**a**, **b**, **e**, **f**), and the range of the *p* values are labeled with asterisks. Statistical differences in **c** were calculated with Kruskal–Wallis test (*p* values) and Dunn’s multiple comparisons test (asterisks). Statistical differences between the two cohorts, R vs NR, in **e** were calculated using the Mann–Whitney *U* test. Correlation analysis was done using Spearman’s correlation

The results indicated that the levels of the chemokine ligands, CXCL9, CXCL10 and CXCL11, binding to the CXC family chemokine receptor (CXCR3), were significantly increased in the patients after 1 month of anti-PD1 therapy (NPX values CXCL9: pre 390 vs. 1mo 901, *p* < 0.001, CXCL10: pre 1000 vs. 1mo 2080, *p* = 0.03, CXCL11: pre 45 vs. 1mo 71, *p* = 0.01, pre 45 vs. 3mo 56, *p* = 0.05; Fig. 4a). Further analysis of these chemokine ligand levels indicated that the increase occurred mainly in the responders after 1 month of anti-PD1 therapy (NPX CXCL9: pre 430 vs. 1mo 1010, *p* = 0.001, CXCL10: pre 1300 vs. 1mo 2600, *p* = 0.03, CXCL11: pre 50 vs. 1mo 78, *p* = 0.03). However, no changes were observed in the non-responders (Fig. 4b).

As the CXC family ligands were known to bind to the CXCR3 receptor and induce lymphocyte tumor infiltration [21–23], we next correlated the CXC ligand serum levels and expression of the CXCR3 receptor in T cells before the initiation of therapy. The phenotype of the PB lymphocytes indicated that the expression of CXCR3 was most frequently expressed in the responders’ CD8^−^CD3^+^ T cells before initiation of therapy when compared to the non-responders and healthy controls (*R*: 71% vs. NR: 49% vs. *H*: 37%, NR vs. *H*, *p* = 0.001) (Fig. 4c). Our results indicated a positive correlation between the pretreatment CXCL9 and CXCR3 expression levels (*r* = 0.58, *p* = 0.02), and a trend between CXCR3 and CXCL10/11 (Fig. 4d).

The Olink assay also indicated heightened levels of other immune cell-stimulating cytokines in the responders’ serum after initiation of anti-PD1 therapy; NK-cell stimulatory factor 2 (IL-12B: pre 34 vs 1mo 46, *p* = 0.01, pre 34 vs. 3mo 42, *p* = 0.009) and tumor necrosis factor receptor superfamily member 9 (TNFRSF9: pre 130 vs. 1mo 170, *p* = 0.03, pre 130 vs. 3mo 160, *p* = 0.02). Moreover, after 3 months of therapy, the responders had higher levels of IL-12B in their sera compared to the non-responders (42 vs. 20, *p* = 0.02, respectively) (Fig. 4e, f). No significant changes were observed in the non-responder serum samples. All serum protein levels at the three studied time points are shown in the supplemental Fig. 2.

### Responders’ tumor harbor granzyme B-expressing CD4^+^CD3^+^T cells

From the findings in the serum protein levels, we sought to further investigate the intratumoral lymphocytes with mIHC. Two representative cases, one responder and non-responder, are shown in supplemental Fig. 3. From the tumor biopsy taken from a well-responding patient, we found a number of CD4^+^ T cells secreting granzyme B (GrB), but little to none were found in the biopsy obtained from the non-responder. However, no statistically significant differences were observed between the biopsy phenotypes of the responders and non-responders.

### Age-associated characteristics may contribute to enhanced therapy response to PD1 inhibition

To find potential biomarkers of response, we further analyzed the differences between responding and non-responding patients before initiation of anti-PD1 therapy.

**Fig. 5:**
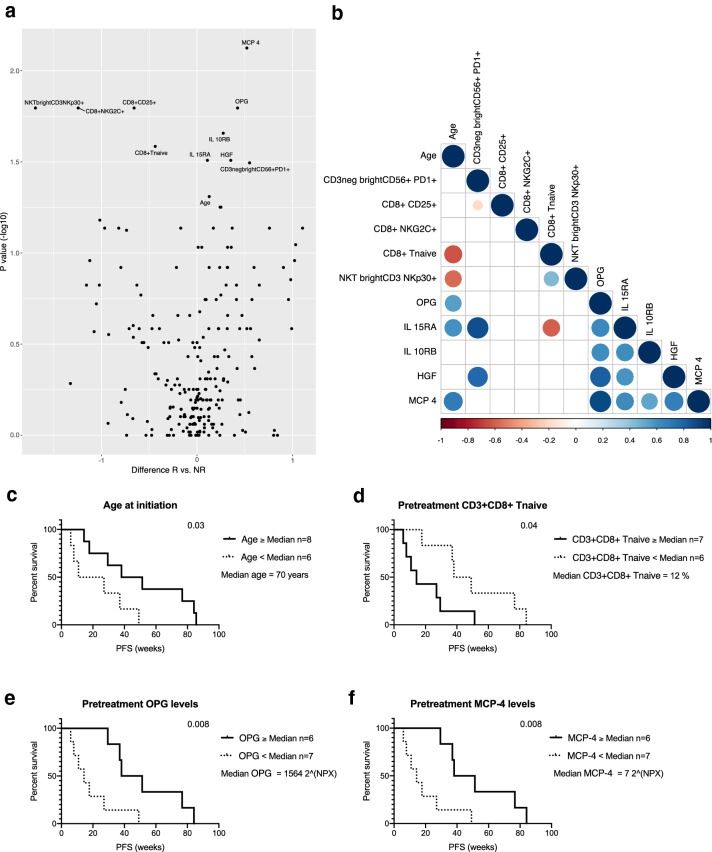
Age-associated factors before initiation of therapy are related to prolonged progression-free survival. **a** Average-weighted absolute difference in median values between responders (R) and non-responders (NR) cohorts at pretreatment visualized in volcano plot, *p* values presented as − log10 scale and a *p* value of 0.05 was set as a threshold for variable labeling, variables 0 < represent higher median in R. **b** Correlation of the significantly different variables between R and NR at pretreatment. Correlation calculated using spearman correlation and *p* value of 0.05 was set as a threshold for variables shown in the plot. The association of these significant variables with PFS was studied using Cox regression analyses and visualized using Kaplan–Meier curves. Patients were grouped into two cohorts as higher or equal (≥) and lower (<) the median **c** age, **d** CD3^+^CD8^+^-naïve T cells, **e** OPG or **f** MCP4 at pretreatment

The average-weighted absolute difference in median values at pretreatment between the responder and non-responder groups using the Mann–Whitney *U* test indicated that the responders had significantly higher levels of serum MCP-4, OPG, IL-10RB, IL-15RA, HGF [difference in median (*d*): *d* = 0.52, *p* = 0.01; *d* = 0.42, *p* = 0.02; *d* = 0.27, *p* = 0.02; *d* = 0.11, *p* = 0.03; *d* = 0.35, *p* = 0.03, respectively), and a higher proportion of PB PD1-expressing CD56^bright^ NK cells (*d* = 0.55, *p* = 0.03). In addition, the responders were significantly older (*d* = 0.13, *p* = 0.05) than the non-responders and had a significantly lower pretreatment proportion of NKp30-expressing CD3^bright^ NKT cells (*d* = − 1.70, *p* = 0.02), NKG2C and CD25-expressing CD8^+^ T cells (*d* = − 0.66, *p* = 0.02, *d* = − 1.24, *p* = 0.02, respectively) and naïve CD8^+^ T cells (*d* = − 0.44, *p* = 0.03) (Fig. 5a).

A correlation analysis was used to further determine the relationships of the different variables. Our analysis indicated that patient age significantly correlated with the proportion of CD8^+^-naïve T cells, NKp30-expressing CD3^bright^ NKT cells and serum OPG, IL-15RA and MCP-4 levels (Spearman correlation: *r* = − 0.61, *p* = 0.01; *r* = − 0.56, *p* = 0.04; *r* = 0.54, *p* = 0.04; *r* = 0.6, *p* = 0.01; *r* = 0.69, *p* = 0.005, respectively) (Fig. 5b).

Furthermore, we studied the age- and treatment response-associated parameters with PFS using Cox regression analysis, by grouping patients into two cohorts as higher or equal (≥) and lower (<) median of each parameter. The analysis indicated that patients who were 70 years or older at the time of initiation of anti-PD1 had significantly longer PFS (HR 0.36 95% CI 0.1–1.3, *p* = 0.03) (Fig. 5c). The lower proportion (< 12% of CD8^+^ T cells) of PB-naïve CD8^+^ T cells before therapy initiation also significantly correlated with longer PFS (HR 0.36, 95% CI 0.1–1.2, *p* = 0.04) (Fig. 5d). In addition, high pretreatment levels of serum OPG and MCP-4 significantly correlated with longer PFS (HR 0.28, 95% CI 0.1–1.0, *p* = 0.008) (Fig. 5e, f).

## Discussion

Novel immune checkpoint inhibitors, such as anti-PD1, have shown great promise in treating solid tumors, especially in metastatic melanoma [13–15]. However, the overall effects of the treatment on the immune system are poorly understood. A deeper understanding of these effects could potentially aid in the discovery of biomarkers and improve patient selection for the treatments. In this study, we hypothesized that NK, NKT and T cells are influenced by PD1 inhibition in metastatic melanoma patients, and that the therapy would potentially affect the immunophenotype. Moreover, we were interested in the pretreatment differences in the PB lymphocytes and plasma protein profiles between the responders and non-responders to identify novel biomarkers of response to anti-PD1 therapy.

Besides T cells, NK and NKT cells are known to have diverse functions in antitumor immune responses by directly killing malignant cells or induce cells such as CD8^+^ T cells to attack the tumor via the immune-activated production of cytokines [16, 24]. Anti-PD1 monoclonal antibodies are designed to inhibit the inactivating binding of the programmed death-ligand 1 (PD-L1), by binding to the PD-1 receptor on the surface of an activated T cell, thus enabling the antitumor immune response by T cells [5]. PD1 expression on NK cells has been previously described in cancer patients and human cytomegalovirus (HCMV) seropositive healthy individuals, and similarly to CD8^+^ T cells, high PD1 expression on NK cells has been characterized as a sign of exhaustion, with lower degranulation or cytotoxic activity and cytokine production compared to PD1^neg^ NK cells [25–28]. Our results were in line with the previous observations that besides PB T cells, PB NK and NKT cells also express PD1 receptors on their surface, supporting our hypothesis that these cells may play an important role in the anti-PD1-mediated antitumor effect for metastatic melanoma patients. The contribution of the PD1 blockade to NK- and NKT-mediated anti-tumor immune responses have previously been shown in murine models [29, 30]; however, no clear evidence of the effects in humans have previously been described.

In this study, we observed that the expressions of CD25 and CD45RO increased in the responders’ cytotoxic CD56^dim^ NK cells after 1 month of anti-PD1 therapy. Previous studies have shown that the expression of CD25 and CD45RO on NK cells have indicated an effector-like phenotype that has been associated with enhanced proliferation, active degranulation and NK-cell-mediated cytotoxicity [31–33]. The CD45RO^+^ NK cells have especially been identified with antitumor activity in hematological cancers [33, 34]; thus, we believe that anti-PD1 therapy could directly or indirectly enhance the cytotoxic potential of NK cells in the responders. Although not significant, we observed that the mean levels of both markers were higher in the responders before treatment, compared to non-responders, suggesting that the responders’ NK cells may already possess a more activated phenotype that is further enhanced with anti-PD1 treatment.

Moreover, we noticed that during anti-PD1 therapy, a proportion of the PB NKT cells increased in responders, and the highest NKT-cell percentage was observed in patients with a complete response to anti-PD1. Interestingly, these complete responders clearly possessed the highest NKT-cell proportion already before initiation of treatment. The mean PB NKT-cell reference value is 5.5% out of the total CD3^+^ [35], and the number of circulating type I NKT cells (invariant NKT cells) have been reported to decrease in different malignancies compared to healthy controls [24]. In addition, Ibarrondo et al. have shown that melanoma patients responding to anti-CTLA4 treatment have high frequencies of circulating NKT cells before and after treatment [36]. Hence, we believe that the high frequency of circulating NKT cells could provide a clinical advantage to melanoma patients treated with anti-PD1, and that the treatment may as well influence the NKT-frequency. However, further studies are needed to determine the exact role of these NKT cells to be utilized as a predictive marker for response.

One potential mechanism of anti-PD1-mediated lymphocyte modulation is the cytokine production pathway. NK and NKT cells are known to produce large amounts of cytokines such as IFNγ, which may further induce the activation of other lymphocytes. Interestingly, we found that the IFNγ-inducible chemokines, CXCL9-11, were significantly increased in the responders after the first month of anti-PD1 therapy. Similarly, Chow et al. recently reported an increase of CXCL9 and CXCL10 in melanoma patients who responded to anti-PD1 therapy. Further, the in vivo* Cxcr3*^*−/−*^ model indicated that the efficacy of anti-PD1-mediated tumor control is dependent on the intratumoral CXCR3 receptor–ligand interaction [37]. The CXCL9-11 chemokines are known to bind CXCR3 and are involved in inducing the infiltration of T and NK cells into the tumor as well as tumor suppression [21–23]. Our results also indicated that high CXCR3 expression on the CD4^+^ T cells correlated with high CXCL9 levels before the initiation of anti-PD1 therapy, and moreover, responders had the highest expression of CXCR3 on the CD4^+^ T cells as well as CXCL9 serum levels.

Subsequently, the observed immunological changes during anti-PD1 therapy mainly occurred in the responders, but not in the non-responders. Moreover, the complete responders often harbored distinct immunological profiles with regards to these variables even before the initiation of therapy. Henceforth, we were interested to find out the pretreatment characteristics that were different between the responders and non-responders. Our results indicated that the responders had distinct cytokine profiles from non-responders, notably MCP-4, OPG, IL-10RB and HGF, which were significantly more abundant in the responders’ sera compared to non-responders. Further, the responders had lower proportions of PB-naïve CD8^+^ T cells and were older in age. Because a decline in the quantity of naïve CD8^+^ T cells is considered as a hallmark of the aging immune system [38], we wanted to study whether the elevated cytokine levels would be associated with age. Interestingly, our results indicated significant correlation of old age with a low proportion of naïve CD8^+^ T cells and elevated levels of OPG and MCP4. In addition to our findings, a previous study by Larsson et al. indicated the association of age with elevated levels of certain serum cytokines, including MCP-4, OPG, IL-10RB and HGF [39].

Aging is an intricate process of gradual deterioration that significantly affects the immune system. Immunosenescence refers to the deterioration of the immune system with aging, affecting the capacity to generate specific immune responses and long-term immune memory. Due to immunosenescence, older patients would in theory, benefit less from immunological therapies [40]. However, emerging evidence suggests that older age might contribute to clinical benefits for patients treated with immune checkpoint inhibitors [41, 42]. The association of age with improved response to anti-PD1 therapy has recently been observed in a large cohort of Danish metastatic melanoma patients; patients between ages 70–80 years had significantly longer OS and PFS compared with younger patients [43]. Furthermore, Perier-Muzet et al. indicated that old age may improve the anti-PD1 response without increasing the risk for severe adverse events [44]. In addition, Kuegel et al. described the positive association between age and anti-PD1 response in melanoma patients, and demonstrated that the tumor microenvironment in older subjects is more favorable for anti-PD1-mediated antitumor response in murine models [45]. Similarly, the correlation of clinical benefit and older age, yet together with multiple age-associated biological variables, was seen in our small cohort of metastatic melanoma patients. Our results indicate that a low frequency of naïve CD8^+^ T cells, and elevated levels of OPG and MCP4 have strong statistical association to older age and prolonged PFS.

**Fig. 6:**
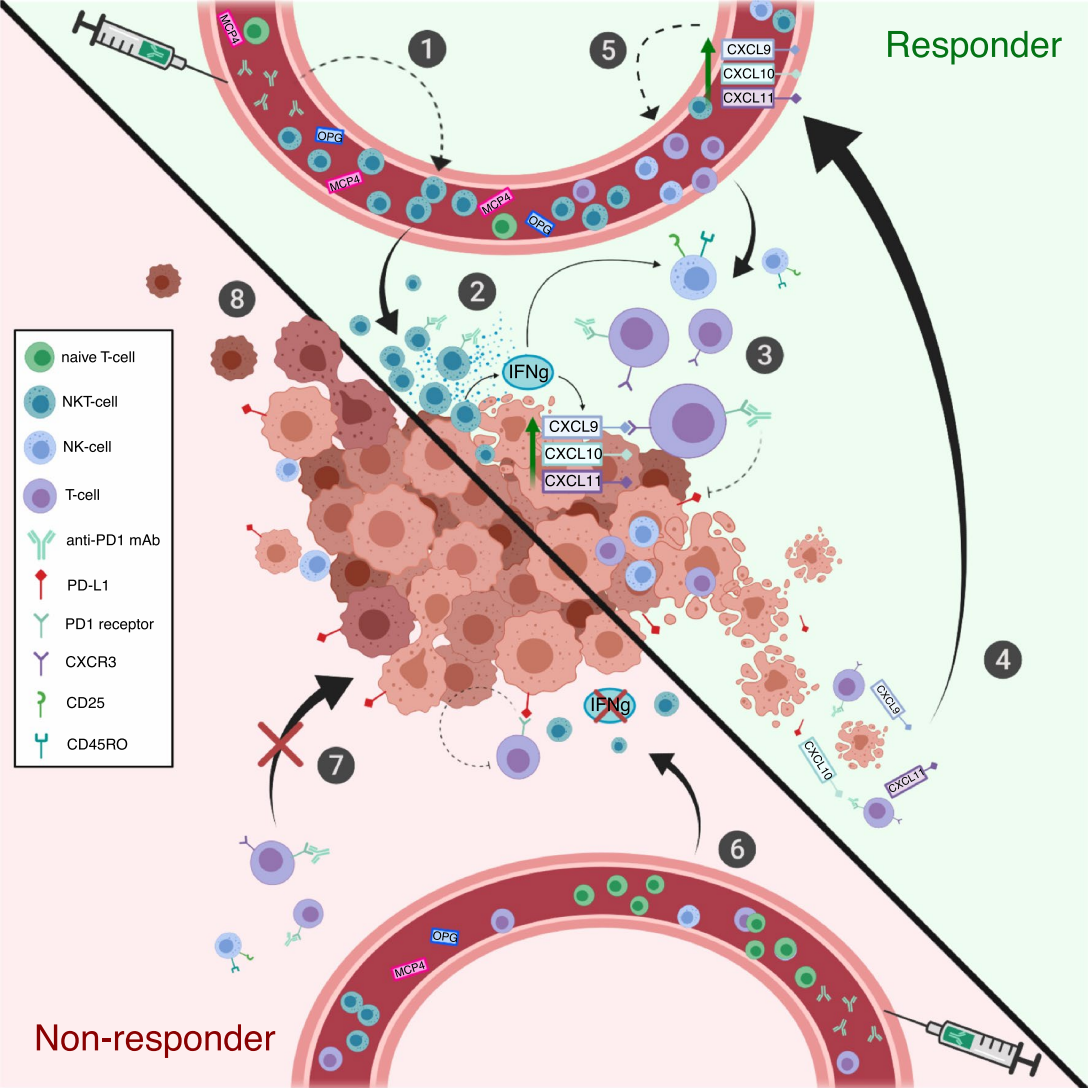
Putative role of observed immunological changes in responders and non-responders. (**1**) The frequency of PB NKT cells is higher and naïve T cells lower in the responders after initiation of anti-PD1 treatment. (**2**) NKT cells are able to produce large quantities of IFNγ, a known activator of other lymphocytes, such as cytotoxic NK- and T cells. IFNγ has also been previously shown to induce the production of CXCL9, -10, and -11 chemokines in tumor microenvironment. (**3**) CXCL9, -10, and -11 are known to induce T-cell migration and infiltration into tumor via CXCR3 interaction. Anti-PD1 treatment blocks the inactivating binding of PD-L1 to its receptor on the surface of cytotoxic T-cell enabling the antitumor immune response. (**4**) CXCR3 ligands (CXCL9, -10, and -11) could be released into circulation as a result of tumor cell lysis, which may further enhance the loop by (**5**) attracting lymphocytes to the tumor site. Further, the responders have increased levels of serum OPG and MCP4, which are cytokines associated with age; however, their role in positive response to anti-PD1 requires further studies. (**6**) Non-responders have less circulating NKT cells, thus the production of IFNγ may be insufficient in activating other lymphocytes and inducing the production of CXCR3 ligands in the tumor microenvironment. (**7**) Without chemokine attraction, the cytotoxic T cells may not be able to migrate to the tumor site, leading to (**8**) insufficient antitumor immune response, tumor growth and disease progression

The comprehensive mechanism of action of the antitumor effect, with the exception of the reactivation of cytotoxic CD8^+^ T cells, in the PD1 blockade is not fully resolved. Additionally, our data suggest that the anti-PD1 treatment increases the frequency of PB NKT cells, the levels of IFNγ-inducible CXC family cytokines and potentially enhances the cytotoxicity of NK cells. We believe that the responders’ NKT cells may play a central role orchestrating the anti-PD1-mediated antitumor immune responses by producing cytokines that direct other lymphocytes to the site of action and activates the cytotoxic effect of these cells. A summary of our results is illustrated in Fig. 6.

As observed, we propose that the age-associated biological characteristics in PB and the increased frequency of NKT cells could potentially contribute to an improved survival for melanoma patients treated with anti-PD1 therapy. This observation needs to be investigated further with larger and prospective clinical immunotherapy trials.

### Electronic supplementary material

Below is the link to the electronic supplementary material.

Supplementary file1 (PDF 22014 kb)

## Abbreviations

CBC: Complete blood count
CR: Complete response
ELR: Eosinophil to lymphocyte ratio
FFPE: Formalin-fixed paraffin embedded
GrB: Granzyme B
IO: Immuno-oncological
mIHC: Multiplexed immunohistochemistry
MLR: Monocyte to lymphocyte ratio
NKT: Natural killer T cell
NLR: Neutrophil to lymphocyte ratio
NPX: Normalized Protein eXpression
NR: Non-responder
PB: Peripheral blood
PD: Progressive disease
PR: Partial response
R: Responder
SD: Stable disease
TMA: Tissue microarray
