# Supplementary material for: Age-associated changes in the immune system may influence the response to anti-PD1 therapy in metastatic melanoma patients
Source: Cancer Immunol Immunother. 2020 Feb 8;69(5):717–30. doi: 10.1007/s00262-020-02497-9 (PMC7183505; doi:10.1007/s00262-020-02497-9)
Supplement: Supplementary file 1 — Supplementary file1 (PDF 22014 kb) [file 262_2020_2497_MOESM1_ESM.pdf]

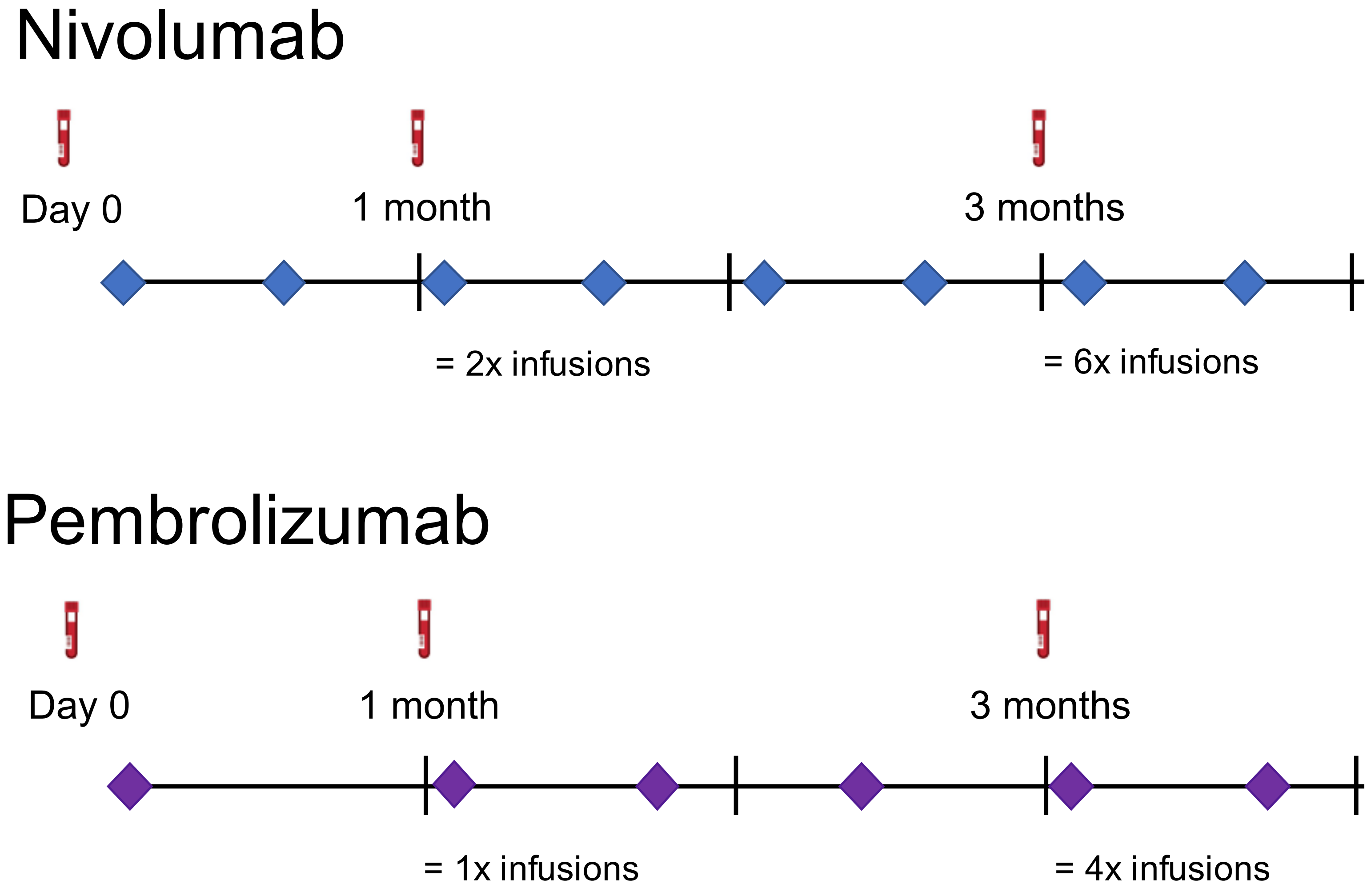


**Supplemental Figure 1.** Blood sampling and drug infusion schedule.

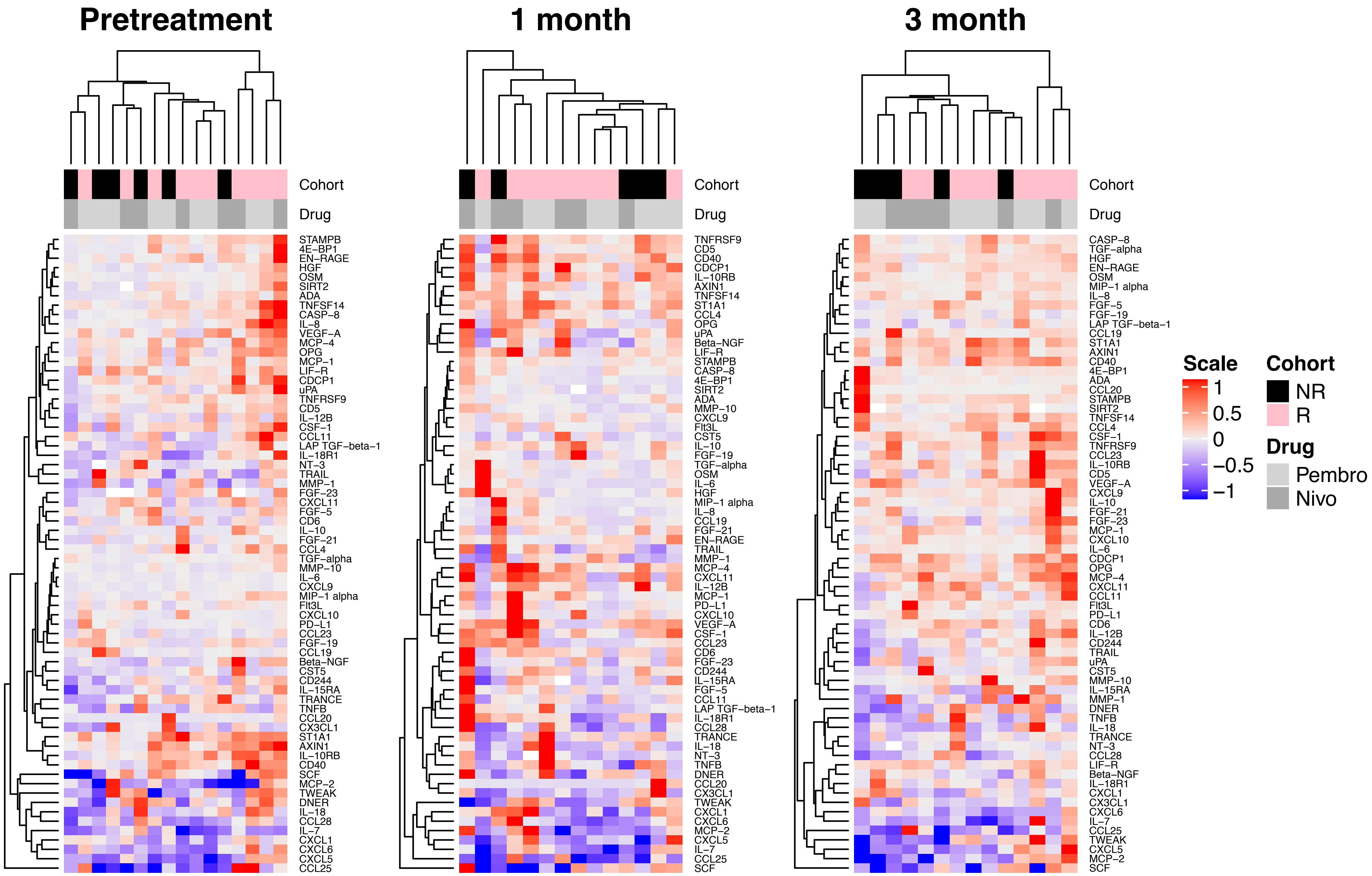


**Supplemental Figure 2.** Serum cytokine profile before and after 1 and 3 months of anti-PD1 therapy. Cohort: NR = non-responder, R = responder.

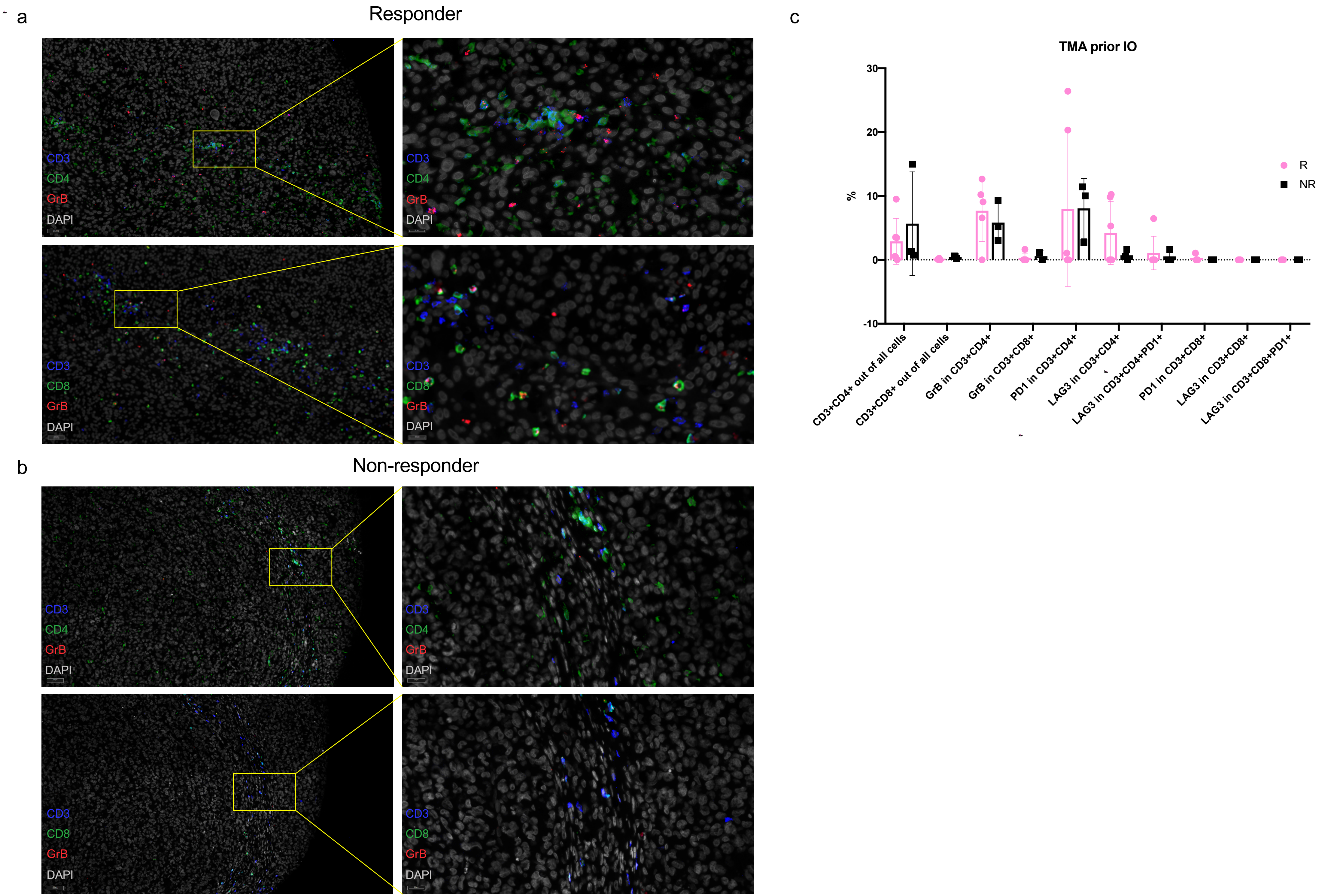


**Supplemental Figure 3.** Multiplexed immunohistochemistry of intratumoral lymphocytes from FFPE metastatic melanoma biopsies at the time of diagnosis. a) Representative case of the TILs from anti-PD1 responder tumor. b) Representative case of the TILs from anti-PD1 non-responder tumor. c) Levels of the TIL subpopulations from all studied biopsies.

| Antibody | Fluorochrome | Catalog no | Manufacturer |
|---|---|---|---|
| CD16 | PE | 561313 | BD |
| CD3 | PerCP-Cy5.5 | 332771 | BD |
| CD4 | PE-Cy7 | 560649 | BD |
| GammaDelta | APC | 555718 | BD |
| CD45 | APC-H7 | 560178 | BD |
| CD8 | BV510 | 563919 | BD |
| CD56 | BV421 | 562751 | BD |
| PD1 (clone: MIH4) | FITC | 557860 | BD |
| PD1 (clone: EH12.1) | BB515 | 564494 | BD |
| LAG-3 | PE | 12-2239-42 | eBioscience |
| ICOS | PE-Cy7 | 25-9948-41 | eBioscience |
| CTLA4 | APC | 560938 | BD |
| HLA-DR | BB515 | 564516 | BD |
| CD27 | PE | 555441 | BD |
| CD25 | PE-Cy7 | 561405 | BD |
| CD11b | APC | 550019 | BD |
| NKG2C | AF488 | FAB138G | RnD |
| CD161 | PE | 556081 | BD |
| NKG2D | PE-Cy7 | 562365 | BD |
| NKG2A | APC | FAB1059A | RnD |
| 3DL1 (CD158e) | FITC | 130-099-387 | Miltenyi Biotec |
| 2DS4 (CD158i) | PE-Vio770 | 130-099-963 | Miltenyi Biotec |
| CD3 | VioGreen | 130-098-164 | Miltenyi Biotec |
| 2DL2/S2/L3 (CD158b) | PE | 130-099-397 | Miltenyi Biotec |
| 2DL1/S1 (CD158a/h) | APC | 130-099-715 | Miltenyi Biotec |
| 2DL1 (CD158a) | APC-Vio770 | 130-103-970 | Miltenyi Biotec |
| CD45 | VioBlue | 130-098-136 | Miltenyi Biotec |
| CD56 | PerCP-Vio700 | 130-100-679 | Miltenyi Biotec |
| DNAM | BB515 | 565152 | BD |
| CD57 | PE | 560844 | BD |
| NKp46 | PE-Cy7 | 562101 | BD |
| NKp30 | APC | 558408 | BD |
| CXCR3 | AF488 | 561730 | BD |
| CCR7 | PE | FAB197P | RnD |
| CD45RO | PE-Cy7 | 560608 | BD |
| CXCR4 | APC | 560936 | BD |

**Supplemental Table 1.** Antibodies used in immunophenotyping assay.
